# Identifying high-risk population of depression: association between metabolic syndrome and depression using a health checkup and claims database

**DOI:** 10.1038/s41598-022-22048-9

**Published:** 2022-11-03

**Authors:** Takahiro Imaizumi, Takuya Toda, Michitaka Maekawa, Daisuke Sakurai, Yuta Hagiwara, Yasuko Yoshida, Masahiko Ando, Shoichi Maruyama

**Affiliations:** 1 Department of Advanced Medicine, Nagoya University Hospital, 65 Tsuruma-cho, Showa-ku, Nagoya, Aichi 464-8550 Japan; 2 Department of Nephrology, Nagoya University Graduate School of Medicine, 65 Tsuruma-cho, Showa-ku, Nagoya, Aichi 464-8550 Japan; 3 Prevent Co., Ltd., Nagoya, Japan; 4 Hidamari Kokoro Clinic, Ama, Japan; 5 Innovative Research Center for Preventive Medical Engineering, Nagoya University, Nagoya, Japan

**Keywords:** Diseases, Medical research, Risk factors

## Abstract

Depression and metabolic syndrome (MetS) are correlated, leading to an increased healthcare burden and decreased productivity. We aimed to investigate the association between MetS-related factors and depression using a health checkup and claims database. Individuals aged 18–75 years who underwent health examinations between 2014 and 2019 were enrolled in the study. Among 76,277 participants, “ever” and “incident” antidepressant users exhibited worse metabolic profiles and were more likely to be prescribed hypnotics and anxiolytics than “never” users. In a nested case–control study with a 1:10 ratio of incident users to controls, MetS was associated with incident antidepressant use (odds ratio, 1.53 [95% confidence interval 1.24–1.88]) adjusted for lifestyle information obtained from a self-administered questionnaire, medical history, and medications. Other metabolic traits also showed significant associations: body mass index (1.04 [1.02–1.06]), abdominal circumference per 10 cm (1.17 [1.08–1.27]), high blood pressure (1.17 [1.00–1.37]), glucose intolerance (1.29 [1.05–1.58]), and dyslipidemia (1.27 [1.08–1.51]). A bodyweight increase > 10 kg from age 20 years (1.46 [1.25–1.70]) was also significantly associated with incident antidepressant use. In conclusion, metabolic abnormalities were associated with incident antidepressant use and can be useful in identifying populations at high risk of depression.

## Introduction

Depression is one of the most common mental illnesses affecting working-age adults and is a major public health problem worldwide^1^. Depression is associated with increased mortality in the general population and among persons with comorbidities^2–5^. A growing body of evidence demonstrates that depression is associated with an increased risk of physical disease, including diabetes mellitus^6,7^ and cardiovascular disease (CVD)^2,8–10^. Furthermore, mental disorders including depression account for approximately 20–50% of disability benefits across the Organisation for Economic Co-operation and Development countries^11^ and have been found to significantly reduce the ability of people to work by increasing other disabilities, including physical ailments^12,13^. Thus, depression not only has the potential to increase net healthcare costs for the treatment of various physical illnesses in addition to depression, but can also cause presenteeism and absenteeism, which potentially lead to lost productive time in the workplace^14,15^.

Various comorbidities, such as cancer, stroke, and diabetes mellitus, are associated with the incidence of depression^16–18^. A systematic review revealed that depression is two to three times more likely to occur in people with multimorbidity than in those without multimorbidity or those with no chronic physical condition. Metabolic syndrome (MetS) is a pre-symptomatic state and is defined as a combination of abdominal or visceral obesity, hypertension, dyslipidemia, and glucose dysregulation, and it has a diagnostic significance in predicting subsequent coronary artery disease, metabolic diseases, and certain cancers^19,20^. Previous studies showed that even MetS is associated with the development of depression^21,22^. However, few longitudinal studies have demonstrated the association between MetS and depression, and knowledge regarding the association between lifestyle and depression remains limited.

In Japan, universal health screening and health guidance were initiated in the early 2000s with the main objective of detecting high-risk populations for CVD^23^. Since then, the word “*metabo*” has been coined in Japan, and this health checkup has become more widespread and promoted. However, one study showed that the effectiveness of prevention of CVD and improvement of anthropometric metrics was limited^24^. Although this is different from the original purpose of this health checkup, if the data from this checkup can be used to show an association between MetS and depression, it may be possible to identify people at high risk of developing depression and add new significance to universal health screening and health guidance.

Therefore, this study aimed to examine the association between MetS-related factors and the development of depression after adjustment for various factors such as medication, prior hospitalization, and lifestyle. We used health insurance claims and health checkup data of corporate insurance beneficiaries, who are representative of the major workforce in Japan. These data covered medical costs, hospitalizations, disease codes, and prescription medicines, allowing us to adjust for comprehensive factors related to the medical interventions received by study participants.

## Results

### Baseline characteristics of the overall study population

**Figure 1:**
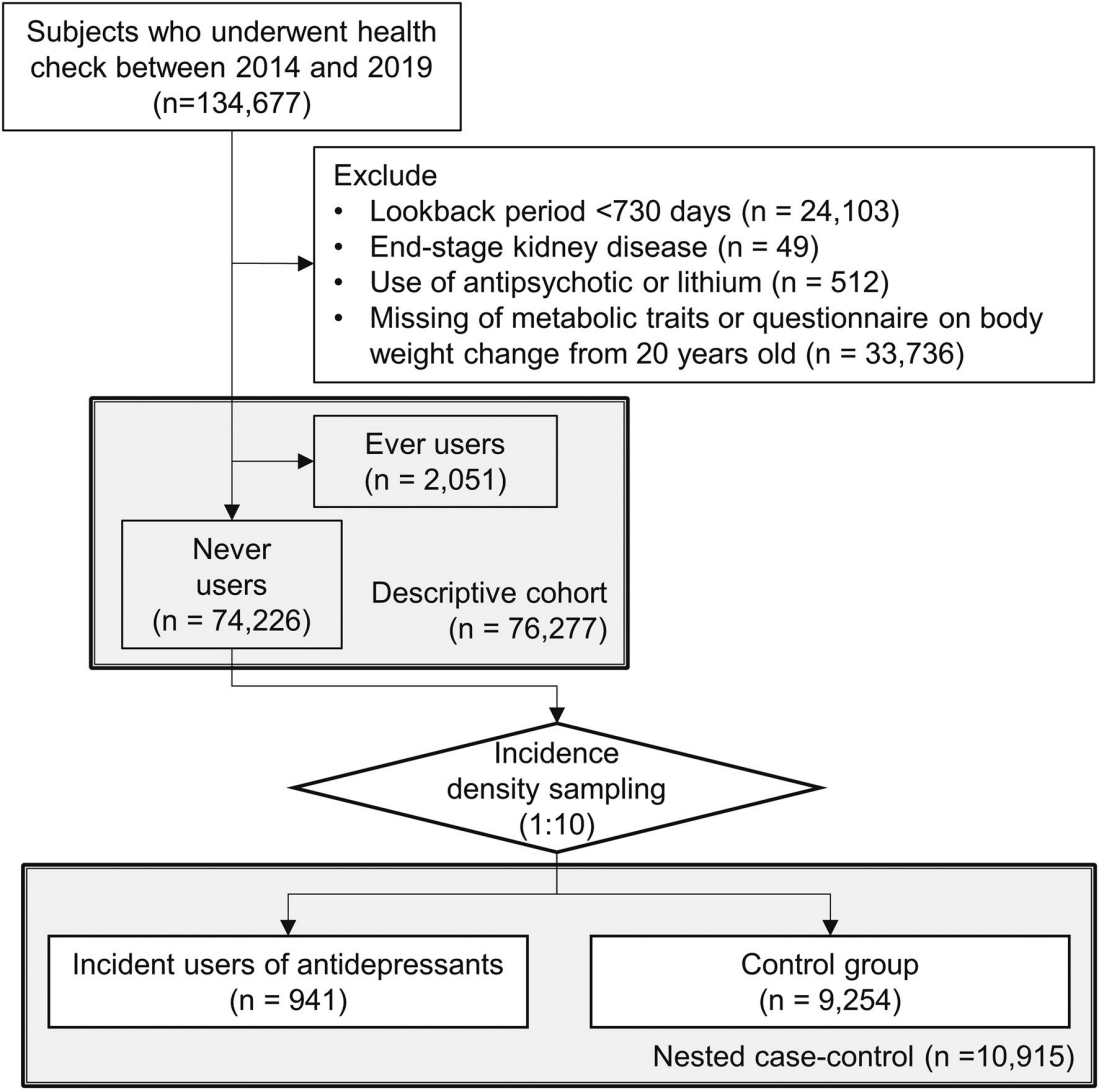
Flow diagram of study subject selection. Flow diagram of study subject selection. The descriptive cohort includes both ever and never users of antidepressants (N = 76,277). The nested case–control study was conducted at a 1:10 ratio of incidence density sampling from never users of antidepressants (N = 10,915).

Individuals aged 18–75 years who underwent health examinations between April 1, 2014, and March 31, 2019, were enrolled in the study. The flow diagram shows the study population and overall study design (Fig. 1). Participants with the following characteristics were excluded: a lookback period < 730 days (n = 33,299); end-stage kidney disease (n = 49); use of second generation antipsychotics or lithium (n = 512); missing information on the following metabolic traits: body mass index (BMI), blood pressure (BP), or abdominal circumference (AC); missing laboratory data on hemoglobin A1c (HbA1c), fasting blood glucose, triglycerides (TG), high-density lipoprotein cholesterol (HDL-c), or low-density lipoprotein cholesterol (LDL-c); or missing questionnaire on bodyweight change from age 20 (n = 33,736). In the descriptive cohort, 76,277 individuals were eligible for the study, including 2051 individuals who had ever used or were currently using antidepressants, 74,226 who had never used antidepressants, and 941 incident users of antidepressants after baseline.

The baseline characteristics of “ever”, “never”, and “incident” users of antidepressants in the descriptive cohort are shown in Table 1. Ever users had a higher proportion of diabetes mellitus and dyslipidemia cases than the other users. Ever and incident users had a high proportion of MetS cases. Questionnaires on lifestyle revealed that never users were less likely to experience bodyweight increase and poor sleep and more likely to be physically active. Late supper, skipping breakfast, and eating speed were not clinically different across the groups. The questionnaire on bodyweight change showed that ever and incident users were more likely to experience bodyweight increase from the age of 20. Regarding alcohol-drinking habits, never users had a lower proportion of occasional or daily drinkers than the others.

**Table 1:** Baseline characteristics of the descriptive cohort.

	N	Total (76,277)	Never users (73,285)	Incident users (941)	Ever users (2051)
Age	76,277	47 (9)	47 (9)	47 (8)	46 (7)
Sex (male)	76,277	46,927 (61.5)	44,864 (61.2)	630 (67.0)	1433 (69.9)
**Metabolic traits**					
Metabolic syndrome	76,277	9083 (11.9)	8602 (11.7)	151 (16.0)	330 (16.1)
Abdominal circumference	76,277	82 (10)	82 (10)	83 (10)	83 (10)
BMI	76,277	23.2 (3.7)	23.2 (3.7)	23.8 (4.0)	23.8 (3.9)
Systolic blood pressure	76,277	121 (16)	121 (16)	122 (16)	121 (15)
Diastolic blood pressure	76,277	75 (12)	75 (12)	76 (11)	76 (11)
Triglyceride	76,277	84 (59–126)	84 (59–125)	93 (63–137)	94 (66–145)
HDL cholesterol	76,277	63 (52–75)	63 (52–75)	60 (50–71)	61 (51–73)
LDL cholesterol	76,276	120 (101–141)	120 (101–141)	121 (102–142)	124 (104–145)
HbA1c	60,169	5.5 (5.3–5.7)	5.5 (5.3–5.7)	5.5 (5.3–5.8)	5.5 (5.3–5.8)
Fasting blood glucose	76,277	94 (87–101)	94 (87–101)	95 (88–103)	95.0 (88–103)
**Self-administered lifestyle information**					
Exercise > 30 min/day	75,063	14,437 (19.2)	13,941 (19.3)	165 (17.9)	331 (16.4)
Physical activity > 60 min/day	74,931	33,593 (44.8)	32,321 (44.9)	411 (44.5)	861 (42.7)
Fast walking speed	28,229	13,147 (46.6)	12,777 (46.7)	115 (42.0)	255 (43.1)
Eating speed	74,924				
Quicker		17,688 (23.6)	17,095 (23.7)	193 (20.9)	400 (19.9)
Normal		44,276 (59.1)	42,546 (59.1)	546 (59.2)	1184 (58.8)
Late		12,960 (17.3)	12,346 (17.2)	183 (19.8)	431 (21.4)
Late supper	74,934	26,041 (34.8)	24,940 (34.6)	353 (38.2)	748 (37.1)
Skipping breakfast	69,302	10,611 (15.3)	10,115 (15.2)	151 (17.7)	345 (18.1)
Poor sleep	74,901	25,780 (34.4)	24,511 (34.1)	429 (46.6)	840 (41.6)
Drinking habits	75,476				
Rarely drink		34,132 (45.2)	32,660 (45.0)	459 (49.5)	1013 (50.0)
Sometimes		23,498 (31.1)	22,659 (31.2)	265 (28.6)	574 (28.3)
Everyday		17,846 (23.6)	17,202 (23.7)	203 (21.9)	441 (21.7)
Current smoking	76,176	17,758 (23.3)	16,954 (23.2)	258 (27.4)	546 (26.7)
> 10 kg increase in BW from age 20	76,277	28,488 (37.3)	27,094 (37.0)	428 (45.5)	966 (47.1)
**Current and past medical history**					
Hypertension	76,277	16,407 (21.5)	15,694 (21.4)	222 (23.6)	491 (23.9)
Diabetes mellitus	76,277	4794 (6.3)	4555 (6.2)	70 (7.4)	169 (8.2)
Dyslipidemia	76,277	27,435 (36.0)	26,184 (35.7)	354 (37.6)	897 (43.7)
Self-reported stroke	75,430	335 (0.4)	321 (0.4)	4 (0.4)	10 (0.5)
Self-reported IHD	75,428	793 (1.1)	752 (1.0)	9 (1.0)	32 (1.6)

Data are expressed as N (%) for categorical values and mean (standard deviation) for continuous values.

*BMI* body mass index, *SBP* systolic blood pressure, *DBP* diastolic blood pressure, *HDL* high-density lipoprotein, *LDL* low-density lipoprotein, *BW* body weight, *IHD* ischemic heart disease, *CVD* cardiovascular disease, *CHF* congestive heart failure, *NSAIDs* non-steroidal anti-inflammatory drugs.

**Figure 2:**
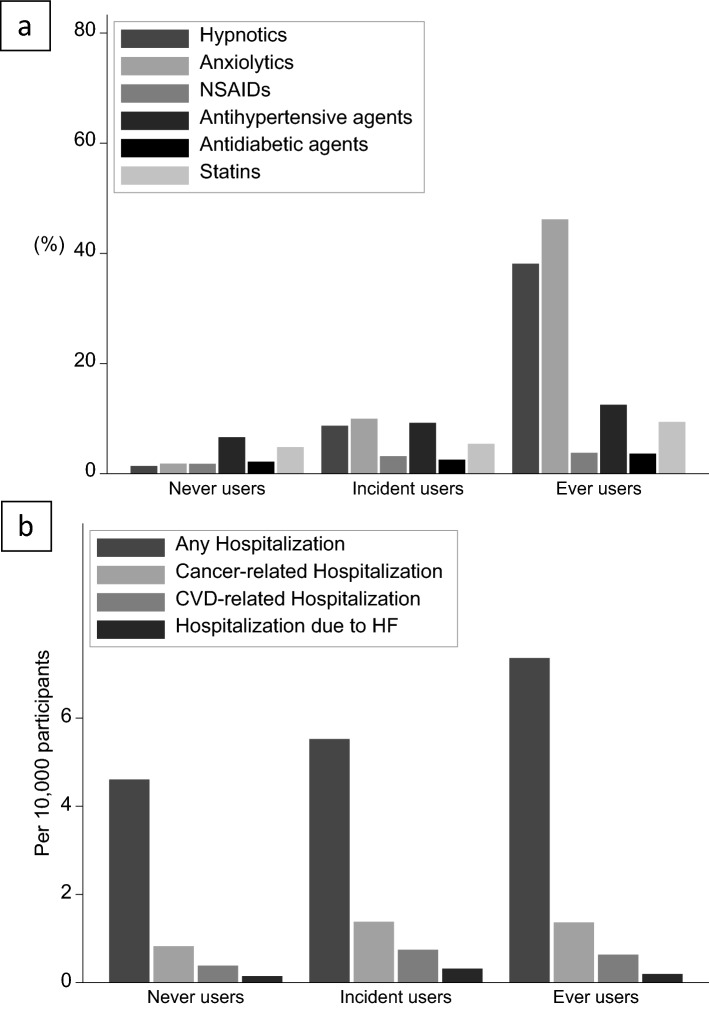
Medication and hospitalization of never, ever, and incident users of antidepressants. Medication and hospitalization of never, ever, and incident users of antidepressants. *NSAIDs* non-steroidal anti-inflammatory drugs, *CVD* cardiovascular disease, *HF* heart failure.

Regarding medication, incident users were more likely to be prescribed hypnotics and analgesics than never users; furthermore, ever users were considerably more likely to be prescribed hypnotics than incident users (Fig. 2a). Regarding hospitalization records, ever and incident users were more likely to be hospitalized, especially cancer-related hospitalization (Fig. 2b).

### Nested case–control study to examine the factors associated with incident antidepressant use

After excluding ever users of antidepressants from the descriptive cohort, we performed a nested case–control study to examine the factors associated with incident antidepressant use. The nested case–control design, also known as risk-set sampling, is a type of study design that identifies controls from a group of people who are “at risk” at the index date of the case^25^. Herein, we defined the day of the first filling of an antidepressant prescription as the index date. For each case, up to 10 controls were selected, and they were selected from subjects of the same sex and age ± 3 years; hence, we finally analyzed the data of 10,915 individuals, including 941 incident users and 9254 controls.

The baseline characteristics are compared in Table 2. Incident users had a higher proportion of MetS cases and hypnotic and analgesic users, and they were more likely to experience body weight increase from age 20 and any hospitalization and cancer-related hospitalizations prior to the index date. Table 3 shows the results of both univariable and multivariable conditional logistic regression analyses to examine associations with incident antidepressant use (case). MetS was significantly associated with the incident antidepressant use in Model 3 (adjusted odds ratio, 1.53 [95% confidence interval 1.24–1.88]). Lifestyle, eating speed, poor sleep, and drinking habits were significantly associated with the outcome. Hospitalization due to cancer and use of hypnotics and anxiolytics were also significantly associated with the outcome. Regarding other metabolic traits, BMI, AC, high BP, glucose intolerance, dyslipidemia, a > 10 kg increase in bodyweight from age 20, and the number of metabolic components were also significantly associated with antidepressant initiation (Table 4). Here, the number of metabolic components was defined as the number of cases with high BP, glucose intolerance, and dyslipidemia.

**Table 2:** Baseline characteristics of the risk-set sample.

	N	Total N = 10,195	Incident users N = 941	Controls N = 9254	P value
Age, years	10,195	47 (7)	47 (8)	47 (7)	0.85
Sex (male)	10,195	6857 (67.3)	630 (67.0)	6227 (67.3)	0.83
**Metabolic traits**					
Metabolic syndrome	10,195	1231 (12.1)	151 (16.0)	1080 (11.7)	< 0.001
Abdominal circumference, cm	10,195	82 (10)	83 (10)	82 (10)	0.001
BMI, kg/m^2^	10,195	23.4 (3.7)	23.8 (4.0)	23.4 (3.7)	< 0.001
SBP, mmHg	10,195	122 (16)	122 (16)	122 (16)	0.69
DBP, mmHg	10,195	76 (12)	76 (11)	76 (12)	0.89
Triglyceride, mg/dL	10,195	88 (60–132)	93 (63–137)	87 (60–131)	0.019
HDL cholesterol, mg/dL	10,195	62 (52–74)	60 (50–71)	62 (52–74)	< 0.001
LDL cholesterol, mg/dL	10,195	121 (103–143)	121 (102–142)	121 (103–143)	0.85
HbA1c, %	8052	5.5 (5.3–5.7)	5.5 (5.3–5.8)	5.5 (5.3–5.7)	0.017
Fasting blood glucose, mg/dL	10,195	94 (88–101)	95 (88–103)	94 (87–101)	< 0.001
**Self-administered lifestyle information**					
Exercise > 30 min/day	10,045	1874 (18.7)	165 (17.9)	1709 (18.7)	0.51
Physical activity > 60 min/day	10,017	4193 (41.9)	411 (44.5)	3782 (41.6)	0.084
Fast walking speed	4123	1909 (46.3)	115 (42.0)	1794 (46.6)	0.14
Eating speed	10,008				< 0.001
Quicker		2926 (29.2)	193 (20.9)	2733 (30.1)	
Normal		5667 (56.6)	546 (59.2)	5121 (56.4)	
Late		1415 (14.1)	183 (19.8)	1232 (13.6)	
Late supper	10,016	3669 (36.6)	353 (38.2)	3316 (36.5)	0.29
Skipping breakfast	9591	1736 (18.1)	151 (17.7)	1585 (18.1)	0.74
Poor sleep	10,006	3632 (36.3)	429 (46.6)	3203 (35.3)	< 0.001
Drinking habits	10,070				< 0.001
Rarely drink		4305 (42.8)	459 (49.5)	3846 (42.1)	
Sometimes		3171 (31.5)	265 (28.6)	2906 (31.8)	
Everyday		2594 (25.8)	203 (21.9)	2391 (26.2)	
Current smoking	10,181	2657 (26.1)	258 (27.4)	2399 (26.0)	0.32
> 10 kg increase in BW from age 20	10,195	4016 (39.4)	428 (45.5)	3588 (38.8)	< 0.001
**Current and past medical history**					
Hypertension	10,195	2274 (22.3)	222 (23.6)	2052 (22.2)	0.32
Diabetes mellitus	10,195	564 (5.5)	70 (7.4)	494 (5.3)	0.007
Dyslipidemia	10,195	3706 (36.4)	354 (37.6)	3352 (36.2)	0.40
Self-reported stroke	10,072	55 (0.5)	4 (0.4)	51 (0.6)	0.62
Self-reported IHD	10,073	71 (0.7)	9 (1.0)	62 (0.7)	0.31
**Hospitalization prior to the index date**					
Any cause	10,195	758 (7.4)	88 (9.4)	670 (7.2)	0.019
CVD	10,195	68 (0.7)	11 (1.2)	57 (0.6)	0.047
CHF	10,195	37 (0.4)	5 (0.5)	32 (0.3)	0.37
Cancer	10,195	137 (1.3)	23 (2.4)	114 (1.2)	0.002
**Medication**					
Hypnotics	10,195	236 (2.3)	82 (8.7)	154 (1.7)	< 0.001
Anxiolytics	10,195	301 (3.0)	94 (10.0)	207 (2.2)	< 0.001
Antihypertensive agents	10,195	586 (5.7)	87 (9.2)	499 (5.4)	< 0.001
Antidiabetic agents	10,195	169 (1.7)	24 (2.6)	145 (1.6)	0.024
NSAIDs	10,195	217 (2.1)	30 (3.2)	187 (2.0)	0.018
Statins	10,195	424 (4.2)	51 (5.4)	373 (4.0)	0.042

Data are expressed as N (%) for categorical values and mean (standard deviation) for continuous values.

*BMI* body mass index, *SBP* systolic blood pressure, *DBP* diastolic blood pressure, *HDL* high-density lipoprotein, *LDL* low-density lipoprotein, *BW* body weight, *IHD* ischemic heart disease, *CVD* cardiovascular disease, *CHF* congestive heart failure, *NSAIDs* non-steroidal anti-inflammatory drugs.

**Table 3:** Factors associated with the initiation of antidepressants.

	N	Unadjusted model	Model 1	Model 2	Model 3
		OR (95% CI)	OR (95% CI)	OR (95% CI)	OR (95% CI)
Metabolic syndrome	10,195	**1.47 (1.21–1.78)**	**1.51 (1.23–1.85)**	**1.49 (1.21–1.83)**	**1.53 (1.24–1.88)**
**Self-administered lifestyle information**					
Exercise > 30 min/day	10,045	0.94 (0.79–1.13)	1.08 (0.89–1.30)	1.08 (0.89–1.31)	1.05 (0.87–1.28)
Eating speed	9821				
Quicker		**0.64 (0.54–0.76)**	**0.63 (0.52–0.75)**	**0.63 (0.52–0.75)**	**0.64 (0.53–0.77)**
Normal		Reference	Reference	Reference	Reference
Late		**1.41 (1.17–1.68)**	**1.48 (1.23–1.78)**	**1.48 (1.23–1.78)**	**1.45 (1.20–1.75)**
Late supper	10,016	1.10 (0.95–1.28)	1.10 (0.94–1.29)	1.10 (0.94–1.30)	1.10 (0.93–1.29)
Skipping breakfast	9591	0.97 (0.80–1.18)	0.97 (0.80–1.18)	0.97 (0.80–1.19)	0.99 (0.81–1.21)
Poor sleep	10,006	**1.60 (1.39–1.83)**	**1.52 (1.31–1.75)**	**1.52 (1.31–1.76)**	**1.42 (1.22–1.64)**
Drinking habits	10,070				
Rarely drink		Reference	Reference	Reference	Reference
Sometimes		0.86 (0.74–1.00)	**0.75 (0.63–0.90)**	**0.76 (0.64–0.91)**	**0.79 (0.66–0.94)**
Everyday		**0.77 (0.65–0.92)**	**0.67 (0.55–0.82)**	**0.67 (0.55–0.82)**	**0.65 (0.53–0.79)**
Current smoking	10,181	1.09 (0.93–1.28)	1.09 (0.92–1.30)	1.09 (0.91–1.30)	1.11 (0.93–1.33)
**Hospitalization prior to the index date**					
CVD	10,195	1.90 (0.99–3.65)		1.79 (0.85–3.79)	1.85 (0.87–3.93)
Cancer	10,195	**2.05 (1.30–3.23)**		**1.81 (1.08–3.02)**	**1.71 (1.01–2.91)**
**Medication**					
Hypnotics	10,195	**5.66 (4.27–7.51)**			**3.37 (2.40–4.73)**
Anxiolytics	10,195	**5.03 (3.88–6.54)**			**3.58 (2.62–4.89)**
NSAIDs	10,195	1.60 (1.07–2.38)			1.32 (0.84–2.07)

Bolded letters represent P values less than 0.05.

*OR* odds ratio, *CI* confidence interval, *BMI* body mass index, *SBP* systolic blood pressure, *DBP* diastolic blood pressure, *HDL* high-density lipoprotein, *LDL* low-density lipoprotein, *BW* body weight, *IHD* ischemic heart disease, *CVD* cardiovascular disease, *CHF* congestive heart failure, *NSAIDs* non-steroidal anti-inflammatory drugs.

**Table 4:** Associations of metabolic traits and BW increase history with incident use of antidepressants.

Metabolic traits	Model 1		Model 2		Model 3	
	OR (95% CI)	P value	OR (95% CI)	P value	OR (95% CI)	P value
Metabolic syndrome	1.51 (1.23–1.85)	< 0.001	1.49 (1.21–1.83)	< 0.001	1.53 (1.24–1.88)	< 0.001
BMI	1.04 (1.02–1.06)	< 0.001	1.04 (1.02–1.06)	< 0.001	1.04 (1.02–1.06)	< 0.001
Abdominal circumference (/10 cm)	1.17 (1.09–1.27)	< 0.001	1.17 (1.08–1.26)	< 0.001	1.17 (1.08–1.27)	< 0.001
> 10 kg increase in BW from age 20	1.43 (1.24–1.66)	< 0.001	1.42 (1.23–1.65)	< 0.001	1.46 (1.25–1.70)	< 0.001
High blood pressure	1.19 (0.99–1.39)	0.027	1.17 (1.01–1.37)	0.042	1.17 (1.00–1.37)	0.048
Glucose intolerance	1.36 (1.12–1.66)	0.002	1.36 (1.11–1.65)	0.003	1.29 (1.05–1.58)	0.014
Dyslipidemia	1.25 (1.06–1.48)	0.008	1.24 (1.05–1.47)	0.010	1.27 (1.08–1.51)	0.005
Number of metabolic components*	1.19 (1.09–1.29)	< 0.001	1.18 (1.08–1.29)	< 0.001	1.18 (1.08–1.28)	< 0.001

Model 1 was adjusted for regular exercise habits > 30 min a day, eating speed, late supper, skipping breakfast, poor sleep, and, drinking habits. Model 2 was adjusted for Model 1 plus hospitalization due to or associated with CVD, and cancer. Model 3 was adjusted for Model 2 plus use of hypnotics, anxiolytics, and NSAIDs.

*OR* odds ratio, *CI* confidence interval, *BMI* body mass index, *BW* body weight.

*The number of any of the following metabolic components: blood pressure, dyslipidemia, and glucose intolerance.

As a sensitivity analysis, the same analysis as in Tables 2 and 3 was performed for the lookback period of 1 year; data from 14,337 individuals, including 1330 affected individuals and 13,307 controls, were analyzed (Supplementary Fig. 1). Similar results were obtained in Supplementary Table 1 and 2.

## Discussion

This study clearly demonstrated that MetS was associated with the incident use of antidepressants after adjustment for various medical and lifestyle factors using large-scale real-world data from health insurance claims and health checkups in Japan. The major finding of the present study was that MetS, other metabolic abnormalities, and pre-symptomatic conditions, including BMI, AC, high BP, glucose intolerance, and dyslipidemia, were associated with antidepressant initiation. We also showed that bodyweight increase of > 10 kg from the age of 20 years was associated with the incident use of antidepressants. MetS and its components were associated with the incident use of antidepressants independent of lifestyle, cancer-related or CVD-related hospitalizations, and medications. We also demonstrated a dose–response association between the number of metabolic components and incident antidepressant use. These findings were of particular interest in that the sum of pre-symptomatic conditions alone was associated with the development of depression despite the absence of a need for disability acceptance or problems with life dysfunction.

This study allowed us to add more significance to health checkups: for screening individuals at high risk of depression. In Japan, a survey showed that the lifetime prevalence and 12-month prevalence of major depressive disorder were 6.1% and 2.2%, respectively, and that the proportion of individuals receiving any type of treatment was 38.7%, which was lower than that in many other high-income countries^26^. The survey found that over 70% of people have moderate or severe depression, yet the low percentage receiving appropriate treatment is problematic. Now that this study showed that MetS is a risk factor for depression, we expect that health checkups and specified health guidance will have a new role as screening sessions for depression and will increase public awareness of the opportunity for early consultation and early appropriate treatment for depression.

The pathways from MetS to depression could be biological or social^27^, a phenomenon that can be explained by the physiological consequences of obesity, including higher inflammation^28,29^ and the psychological/social consequences of MetS or obesity. However, most of the health-related behaviors assessed by the questionnaire, such as exercise habits, late supper, skipping breakfast, and smoking, were not significantly different between the cases and controls. Notably, information on physical activity or exercise habits was collected using self-administered questionnaires rather than quantitative methods. More detailed research is required to investigate physical activity and exercise habits more quantitatively and accurately through a quantitative physical activity using biometric sensors and detailed questionnaires on exercise habits.

Regarding psychosocial factors, physical and mental stress can be common causes of MetS and depression. The study subjects were corporate insurance beneficiaries comprising corporate employees and their dependents, and mental stress is potentially caused by the work itself, human relationships in the workplace, and family issues in this population. Physical stress may be caused by shift work. These not only trigger depression, but can also result in obesity and MetS through overeating, sleep disorders, and effects on various metabolic systems. However, this study did not include information such as work shifts and work-related stress, and further studies are required to identify modifiable factors and identify solutions to overcome both health issues.

We also observed interesting findings regarding lifestyle. First, we found that alcohol consumption is potentially protective against depression. Several previous studies have shown consistent results regarding the protective effect of alcohol against depression^30–34^. Although our results did not show a clear benefit of daily physical activity and modifiable lifestyle factors including sleep and exercise habits as potential candidates for intervention in depression and MetS. Daily exercise habits are particularly suitable lifestyle interventions, as a large body of evidence demonstrates the effectiveness of exercise on depression and sleep disorders^35–38^.

Regarding prescription medicine, the use of hypnotics, anxiolytics, and NSAIDs at baseline was associated with incident antidepressant use. Studies have shown that insomnia is associated with depression and anxiety disorders^39,40^. However, depression is often underdiagnosed in primary-care settings or for older patients^41^, and such medications might have been prescribed as supportive care for patients with symptoms and complaints related to depression but not formally diagnosed. Hence, individuals with underdiagnosed and undertreated depression may be hidden among those who have been prescribed these drugs.

Finally, a history of hospitalization for malignant diseases was also associated with incident antidepressant use. However, the association was attenuated after adjusting for medication information. This may be due to the mediation effects of anxiety and insomnia on the association. Previous studies have demonstrated that anxiety, depression, and insomnia are associated with cancer as well as with increased adverse outcomes, including mortality and psychosocial problems in cancer survivors^42–45^. The present study reaffirms the importance of psychological care for those who experience cancer-related hospitalization.

This study had certain limitations. First, the present study was observational and did not demonstrate any causality. However, our results revealed various medical and lifestyle factors associated with depression, which we believe provide valuable insights into workplace mental and physical health. Second, we classified depression based on specific, prescribed classes of antidepressants, that is, selective serotonin reuptake inhibitors (SSRIs), serotonin-noradrenalin reuptake inhibitors (SNRIs), or noradrenergic and specific serotonin antidepressants (NaSSAs), thus potentially leading to misclassification bias since those who were treated with other classes of medication or those who had not been treated with medication were not diagnosed with depression. This type of misclassification is unavoidable in large-scale database studies. However, the main purpose of this study was to explore and generate hypotheses for future research. Therefore, sufficient findings have been generated from this analysis. Third, there was a concern regarding selection bias. Those who were taking sick leave due to depression might not have undergone health checkups in the workplace. However, in our country, the number of individuals who undergo health checkups is high in the working generations. Finally, most of the lifestyle information was derived from self-administered questionnaires, thus potentially undermining accuracy and objectivity. Furthermore, recall bias may have existed when filling out the questionnaire. We may consider using activity monitoring^46^, such as gyrometers, to gain objective personal activity information in future studies.

In conclusion, MetS and other metabolic abnormalities were associated with incident antidepressant use in working-age individuals. This study also allowed us to add more significance to health checkups: for screening individuals at high risk of depression. Lifestyle intervention could be the subsequent step in reducing both mental and physical burdens among individuals in their prime.

## Methods

### Data source and ethical issues

The data source comprised anonymized, processed receipt information and medical checkup data provided by health insurance associations in Japan contracted with PREVENT Co., Ltd, stored at the company. The present study analyzed data from the administrative claims-based database that included information on 134,677 individuals who underwent health examinations at least once and were under health insurance coverage between April, 2014 and March, 2019, targeted at corporate employees and their dependents in Japan. All data was extracted and processed on November 17, 2020. We conducted this study in accordance with the guidelines of the Declaration of Helsinki. The institutional ethics committee of Nagoya University Graduate School of Medicine formally approved this study (Approval No. 2020-0142). The Ethical Review Committee for Observational Research is an officially approved and registered organization (No. 15000226). Since the study data were provided anonymously, and the study participants did not receive any intervention, informed consent for study participation was waived by ethics committee of Nagoya University Graduate School of Medicine.

### Definitions

We obtained the following information from checkup data: sex, age, BMI, BP, and AC data; laboratory data on HbA1c, fasting blood glucose, TG, HDL-c, and LDL-c; smoking and alcohol habits; and lifestyle and behavior, including weight changes, exercise habits, physical activity, walking speed, eating speed and habits, and poor sleep. Blood tests and physical measurements such as BMI, BP, and AC were performed at the nearest local clinic, hospital, or health screening center for each subject, using standard procedures. A habitual cigarette smoker was defined as a person who smoked a total of over 100 cigarettes or for over six months and has smoked in the last month. Exercise habits were defined as exercising to sweat lightly for > 30 min per session, twice weekly, for over a year. Physical activity was defined as walking or performing an equivalent amount of physical activity for > 1 h per day. Fast walking speed was defined as faster speed than that of almost the same age and of the same gender. Eating speed was categorized into the following three categories: “quicker” than others, “normal,” and “late.” Late supper was defined as eating supper 2 h before bedtime more than three times a week. Skipping breakfast was defined as skipping breakfast more than three times a week. Bodyweight change was defined as a > 10 kg increase in body weight from the age of 20 years.

**Figure 3:**
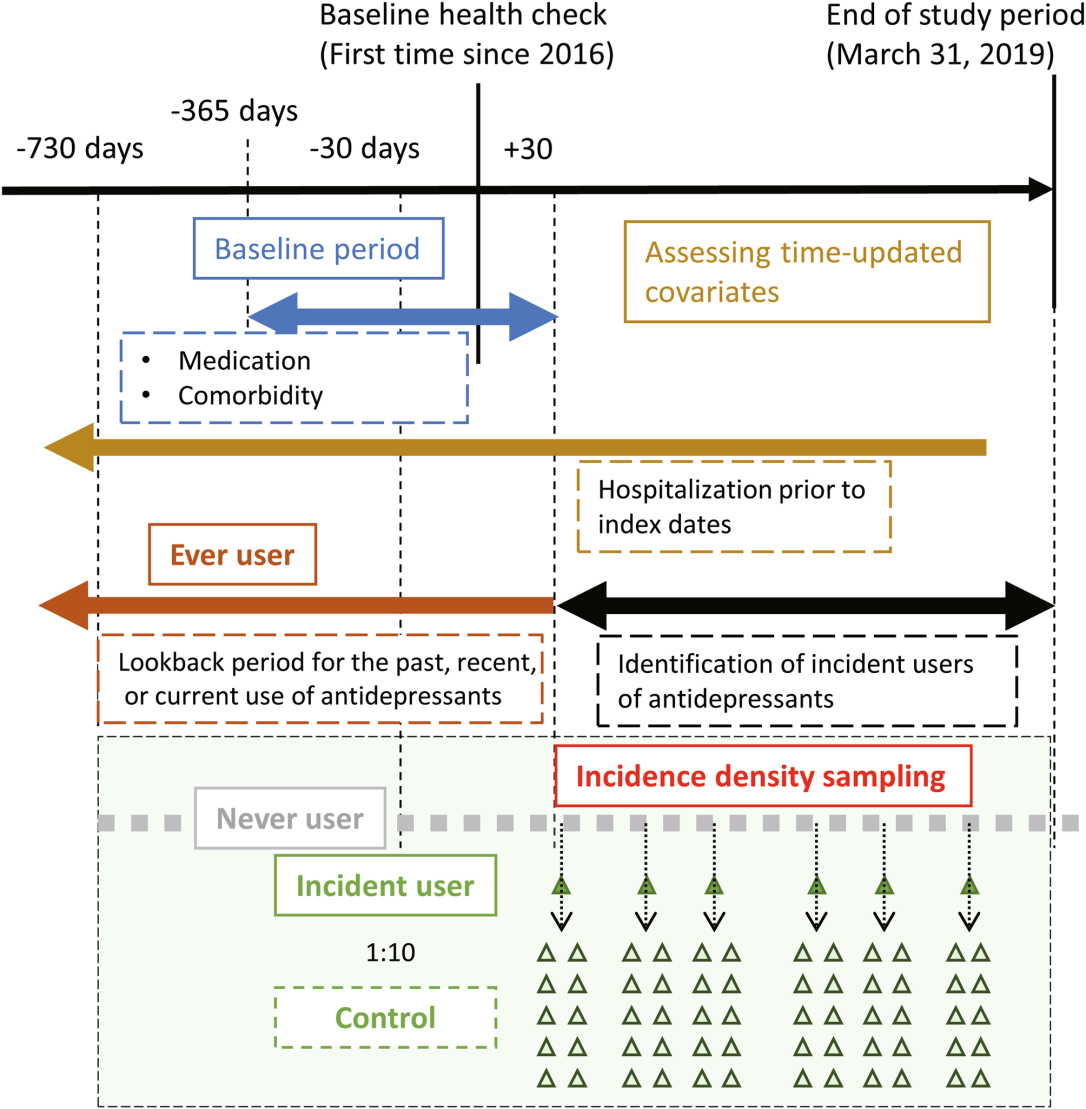
Design diagram of the study. A baseline health check was performed after April 2016. Each case had a lookback period of at least 2 years to assess laboratory data, medication, comorbidity, and hospitalization. Incidence density sampling was performed to conduct a nested case–control study.

We defined the following medicines or their combination as antidepressants: SSRI, SNRI, and NaSSA. We did not include other types of classical antidepressants, including tricyclic or tetracyclic antidepressants, or serotonin antagonists and reuptake inhibitors, since they have been used for purposes other than depression treatment. We defined “ever” users as individuals whose last antidepressant prescription filling was earlier than 30 days after the baseline date and “never” users as those who had never been prescribed an antidepressant within 30 days after the baseline date (Fig. 3). “Incident” users were defined as individuals who had not been prescribed any antidepressants for at least the past 2 years (730 days) and who were initiated on antidepressants > 30 days after the baseline date. In a prior study, the lookback period was set as 1 year^47^. However, the study population comprised youths who were 5 to 20 years of age. Considering the lifelong recurrence of major depressive disorder, we set the lookback period as 2 years^48^. For sensitivity analysis, we set the lookback period as 1 year and performed the same analysis.

Additionally, we obtained baseline data on medication history other than antidepressants, disease name, hospitalization, and procedure from the claims data prior to 30 days after the baseline. A positive medication history was defined as at least one prescription filling during the study period. Data on antihypertensive agents, antidiabetic agents, statins, hypnotics, and NSAIDs were collected using the World Health Organization anatomical therapeutic chemical classification codes (Supplementary Table 2). Detailed information on drug code combinations representing these drugs is available on GitHub (https://github.com/PREVENT-Inc/MyscopeMasterList/tree/master/nagoya-u/medical_conditions_and_depression).

Hospitalization information was also obtained from claims data prior to 30 days after the baseline date. Hospitalizations due to specific causes were defined using a combination of the International Statistical Classification of Diseases and Related Health Problems (ICD)-10 codes in the hospitalization information of medical claims data. Herein, we classified hospitalizations into the following categories: any cause, CVD, congestive heart failure, cancer, and psychotic disorders. CVD-related hospitalization was defined as having any of the following diseases: acute myocardial infarction, congestive heart failure, and cerebrovascular disease. The combinations of ICD-10 codes have been described in a previous study by Quan et al.^49^ Hospitalizations due to mental disorders were classified based on the ICD-10 classification of mental and behavioral disorders^50^.

### Statistical analysis

For between-group comparisons, categorical variables are expressed as numbers and percentages, and continuous variables as the median (interquartile range) or mean (standard deviation).

In the nested case–control study, a between-group comparison was performed in a fashion similar to the comparison of the overall study participants. Univariable and multivariable conditional logistic regression was used to examine the factors associated with the incident prescription of antidepressants. Covariates included age; sex; smoking and drinking habits; physical activity; poor sleep; the use of hypnotics, anxiolytics, and NSAIDs; and hospitalizations due to CVD and cancer. Hospitalization records were utilized in a time-updated manner, implying that hospitalization records were collected not only from baseline but also from data prior to the index dates. Two-sided statistical significance was set at P < 0.05. All analyses were performed using Stata version 17.0 software (StataCorp, TX, USA).

## Supplementary Information

Supplementary Information.

## Data Availability

The data used in this study is the property of PREVENT Co., Ltd. and the purpose of use is restricted by PREVENT’s policy on data utilization. The data used in this study was also used under contract and is not available to the public. Inquiries about the data can be made via GoogleForm [https://forms.gle/iWbhLjEx157dkXZB9], and can available after a license agreement is signed.
