# Supplementary material for: Identifying high-risk population of depression: association between metabolic syndrome and depression using a health checkup and claims database
Source: Sci Rep. 2022 Nov 3;12:18577. doi: 10.1038/s41598-022-22048-9 (PMC9633757; doi:10.1038/s41598-022-22048-9)
Supplement: Supplementary file 1 — Supplementary Information. [file 41598_2022_22048_MOESM1_ESM.pdf]

Supplementary Information

Identifying High-risk population of Depression: Association Between Metabolic Syndrome and Depression Using a Health Checkup and Claims Database

Running title: Metabolic syndrome and depression

Authors:

Takahiro Imaizumi, Takuya Toda, Michitaka Maekawa, Daisuke Sakurai, Yuta Hagiwara, Yasuko Yoshida[5], Masahiko Ando, Shoichi Maruyama

Supplementary Table 1. Factors associated with the initiation of antidepressants (1-year lookback period)

	N	Unadjusted model	Model 1	Model 2	Model 3
		OR (95% CI)	OR (95% CI)	OR (95% CI)	OR (95% CI)
**Metabolic syndrome**	14337	**1.30 (1.10–1.53)**	**1.31 (1.10–1.56)**	**1.30 (1.10–1.56)**	**1.29 (1.08–1.55)**
**Self-administered lifestyle information**					
Exercise >30 min/day	13730	0.94 (0.81–1.11)	1.01 (0.85–1.19)	1.01 (0.85–1.19)	1.02 (0.86–1.20)
Eating speed					
Quicker		**0.67 (0.57–0.79)**	**0.68 (0.58–0.80)**	**0.68 (0.58–0.80)**	**0.71 (0.60–0.84)**
Normal		Reference	Reference	Reference	Reference
Late		**1.22 (1.05–1.42)**	**1.23 (1.06–1.44)**	**1.22 (1.05–1.43)**	**1.24 (1.06–1.45)**
Late supper	13702	1.10 (0.97–1.25)	1.09 (0.95–1.24)	1.09 (0.95–1.25)	1.12 (0.97–1.28)
Skipping breakfast	13063	0.97 (0.83–1.14)	1.00 (0.85–1.44)	1.00 (0.85–1.17)	0.99 (0.84–1.16)
Poor sleep	13709	**1.48 (1.31–1.67)**	**1.44 (1.27–1.63)**	**1.44 (1.27–1.63)**	**1.38 (1.21–1.56)**
Drinking habits	14168				
Rarely drink		Reference	Reference	Reference	Reference
Sometimes		0.91 (0.80–1.03)	**0.80 (0.69–0.93)**	**0.80 (0.69–0.93)**	**0.83 (0.72–0.97)**
Everyday		**0.81 (0.70–0.94)**	**0.72 (0.61–0.86)**	**0.72 (0.61–0.86)**	**0.74 (0.62–0.88)**
Current smoking	14315	0.99 (0.86–1.13)	0.98 (0.85–1.14)	0.98 (0.85–1.14)	1.00 (0.86–1.16)
**Hospitalization prior to the index date**					
CVD	14337	1.66 (0.94–2.95)		1.44 (0.77–2.71)	1.55 (0.82–2.92)
Cancer	14337	**1.98 (1.35–2.89)**		**1.74 (1.13–2.67)**	1.51 (0.97–2.34)
**Medication**					
Hypnotics	14337	**3.59 (2.80–4.59)**			**1.89 (1.40–2.56)**
Anxiolytics	14337	**4.76 (3.86–5.86)**			**3.66 (2.86–4.68)**
NSAIDs	14337	**1.92 (1.41–2.61)**			**1.69 (1.19–2.40)**

Bolded letters represent P values less than 0.05. Abbreviations: OR, odds ratio; CI, confidence interval; BMI, body mass index; SBP, systolic blood pressure; DBP, diastolic blood pressure; HDL, high-density lipoprotein; LDL, low-density lipoprotein; BW, body weight; IHD, ischemic heart disease; CVD, cardiovascular disease; CHF, congestive heart failure; NSAIDs, non-steroidal anti-inflammatory drugs

Supplementary Table 2. Associations of metabolic traits and BW increase history with incident use of antidepressants (1-year look back period)

Metabolic traits	Model 1		Model 2		Model 3	
	OR (95% CI)	P value	OR (95% CI)	P value	OR (95% CI)	P value
Metabolic syndrome	1.31 (1.10–1.56)	0.003	1.30 (1.09–1.55)	0.004	1.29 (1.08–1.55)	0.005
BMI	1.03 (1.01–1.04)	0.002	1.03 (1.01–1.04)	0.002	1.03 (1.01–1.04)	0.002
Abdominal circumference (/10 cm)	1.14 (1.06–1.21)	<0.001	1.13 (1.06–1.21)	<0.001	1.14 (1.06–1.22)	<0.001
>10 kg increase in BW from age 20	1.32 (1.17–1.50)	<0.001	1.32 (1.16–1.50)	<0.001	1.31 (1.15–1.48)	<0.001
High blood pressure	1.12 (0.98–1.28)	0.088	1.11 (0.97–1.27)	0.12	1.13 (0.99–1.30)	0.069
Glucose intolerance	1.24 (1.04–1.49)	0.016	1.24 (1.04–1.49)	0.015	1.26 (1.05–1.50)	0.013
Dyslipidemia	1.10 (0.95–1.27)	0.21	1.10 (0.95–1.27)	0.21	1.06 (0.91–1.22)	0.46
Number of metabolic components	1.11 (1.03–1.19)	0.008	1.10 (1.02–1.19)	0.011	1.10 (1.02–1.19)	0.014

Model 1 was adjusted for regular exercise habits >30 minutes a day, eating speed, late supper, skipping breakfast, poor sleep, and, drinking habits. Model 2 was adjusted for Model 1 plus hospitalization due to or associated with CVD, and cancer. Model 3 was adjusted for Model 2 plus use of hypnotics, anxiolytics, and NSAIDs.

* The number of any of the following metabolic components: blood pressure, dyslipidemia, glucose intolerance

Abbreviations: OR, odds ratio; CI, confidence interval; BMI, body mass index; BW, body weight.

Supplementary Table 3. Medicine list based on ATC classification

Drug class	Subclasses	ATC codes
Antidepressant	SSRI, SNRI, NaSSA	N06
Hypnotics	Benzodiazepine, non-benzodiazepine, orexin receptor antagonists, melatonin receptor agonists	N05C
Anxiolytics	Benzodiazepine	N05B
Antipsychotics	Lithium, multi-acting-receptor-targeted agents, serotonin-dopamine antagonists, Partial Dopamine antagonists, Selective D2/D3 antagonists	N05A
NSAIDs	Non-selective NSAIDs, Selective NSAIDs	M01A<br>N02B
Anti-hypertensive agents	Renin-angiotensin system inhibitors, calcium channel blockers, alpha-blockers, beta-blockers, diuretics, other types of antihypertensive agents	C02, C03, C05, C07, C08, C09
Anti-diabetic agents	SGLT-2 inhibitors, metformin, sulfonylurea, DPP-4 inhibitors,<br>GLP-1RA, glinide, thiazolidine, acarbose, insulins	A10A, A10B
Statins	Statins	C10A

ATC, anatomical therapeutic chemical; SSRI, selective serotonin reuptake inhibitor; SNRI, serotonin noradrenaline reuptake inhibitor; NaSSA, noradrenergic and specific serotonergic antidepressant; NSAIDs, non-steroid anti-inflammatory drugs; SGLT, sodium-glucose co-transporter; DPP-4, dipeptidyl peptidase-4; GLP-1RA, glucagon-like peptide-1 receptor agonists

Supplementary Figure 1. Flow diagram of study subject selection (1-year lookback period)

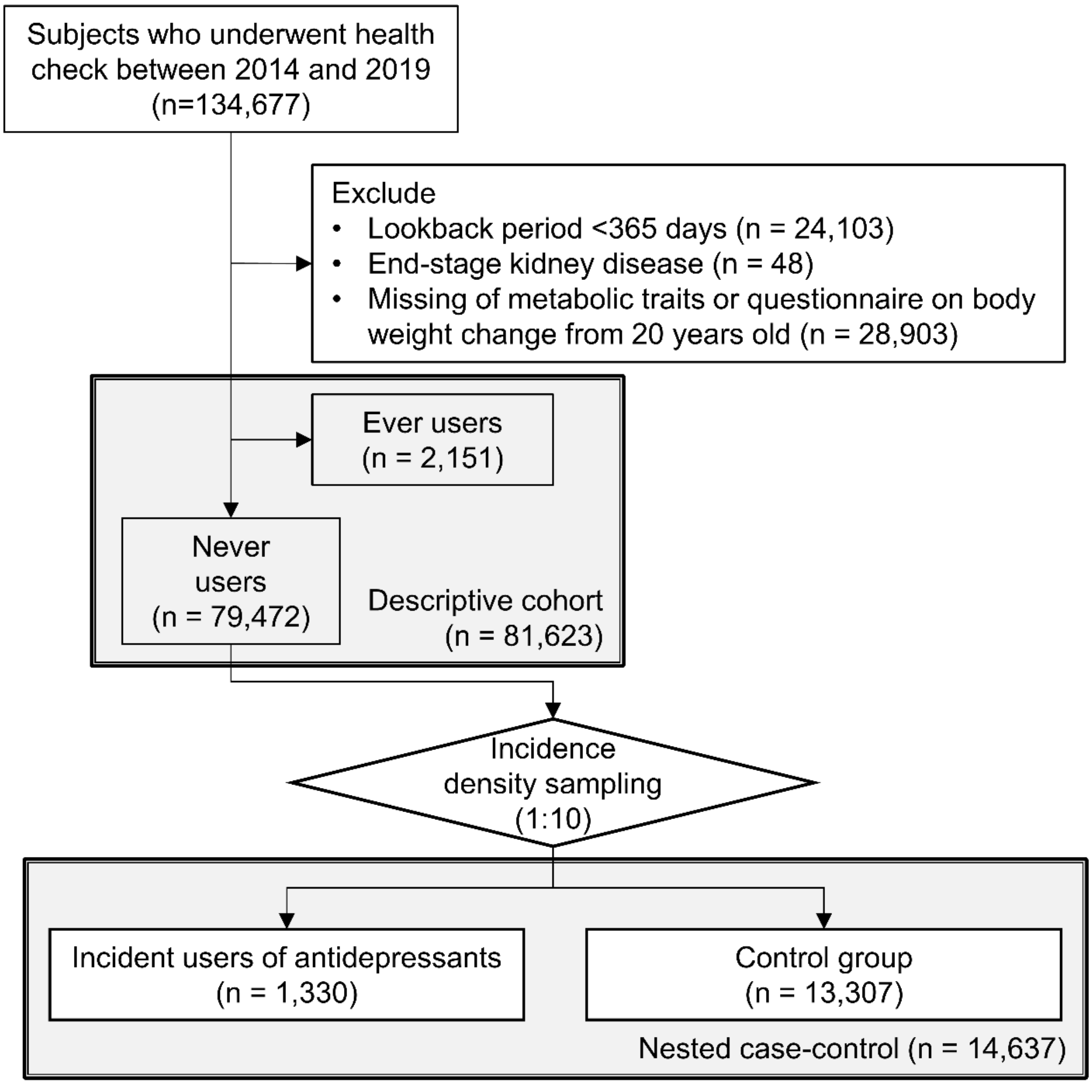

Flow diagram of study subject selection. The descriptive cohort includes both ever and never users of antidepressants (N = 81,623). The nested case-control study was conducted at a 1:10 ratio of incidence density sampling from never-users of antidepressants (N = 14,637).
